# Downregulation of miR-135b-5p Suppresses Progression of Esophageal Cancer and Contributes to the Effect of Cisplatin

**DOI:** 10.3389/fonc.2021.679348

**Published:** 2021-07-01

**Authors:** Yuzhu Di, Yanan Jiang, Xiuyun Shen, Jing Liu, Yang Gao, Huimin Cai, Xiaoli Sun, Dandan Ning, Bing Liu, Jiaji Lei, Shizhu Jin

**Affiliations:** ^1^ Department of Gastroenterology and Hepatology, The Second Affiliated Hospital of Harbin Medical University, Harbin, China; ^2^ Department of Pharmacology (State-Province Key Laboratories of Biomedicine-Pharmaceutics of China, Key Laboratory of Cardiovascular Research, Ministry of Education), College of Pharmacy, Harbin Medical University, Harbin, China; ^3^ Translational Medicine Research and Cooperation Center of Northern China, Heilongjiang Academy of Medical Sciences, Harbin, China; ^4^ Department of Thoracic Surgery, The Second Affiliated Hospital of Harbin Medical University, Harbin, China

**Keywords:** esophageal cancer, miR-135b-5p, thioredoxin interacting protein, cisplatin, proliferation

## Abstract

Esophageal cancer (EC) is one of the commonest human cancers, which accompany high morbidity. MicroRNAs (miRNAs) play a pivotal role in various cancers, including EC. Our research aimed to reveal the function and mechanism of miR-135b-5p. Our research identified that miR-135b-5p was elevated in EC samples from TCGA database. Correspondingly real-time PCR assay also showed the miR-135b-5p is also higher expressed in Eca109, EC9706, KYSE150 cells than normal esophageal epithelial cells (Het-1A). CCK8, Edu, wound healing, Transwell assay, and western blot demonstrated miR-135b-5p inhibition suppresses proliferation, invasion, migration and promoted the apoptosis in Eca109 and EC9706 cells. Moreover, the miR-135b-5p inhibition also inhibited xenograft lump growth. We then predicted the complementary gene of miR-135b-5p using miRTarBase, TargetScan, and DIANA-microT. TXNIP was estimated as a complementary gene for miR-135b-5p. Luciferase report assay verified the direct binding site for miR-135b-5p and TXNIP. Real-time PCR and western blot assays showed that the inhibition of miR-135b-5p remarkably enhanced the levels of TXNIP in Eca109 and EC9706 cells. Furthermore, cisplatin (cis-diamminedichloroplatinum II, DDP) decreased miR-135b-5p expression and increased TXNIP expression. Enhanced expression of miR-135b-5p attenuated the inhibitory ability of cisplatin (cis-diamminedichloroplatinum II, DDP) in Eca109 cells, accompanied by TXNIP downregulation. In conclusion, the downregulation of miR-135b-5p suppresses the progression of EC through targeting TXNIP. MiR-135b-5p/TXNIP pathway contributes to the anti-tumor effect of DDP. These findings may provide new insight into the treatment of EC.

## Introduction

The latest statistics show that esophageal cancer (EC) is the 8th commonest cancer and the 6th commonest reason for cancer-related death worldwide (1). The EC incidence is highest in China, accounting for 54% of all cases (2). The current treatment mainly depends on surgery combined with chemoradiotherapy and immunotherapy. Cisplatin (diamminedichloroplatinum, DDP) is widely used as the first-line chemotherapy approach for different cancers (3, 4). It had been demonstrated an effective therapeutic approach for EC. Lots of researches have confirmed that a variety of miRNAs participated in the therapeutic mechanism of DDP, different miRNAs resist or enhance the therapeutic effect of cisplatin (5, 6). For patients with ESCC, whereas the rate of 5-year survival is also less than 20% (7). Thus, it is imperative to find efficient therapeutic strategies.

MicroRNAs (miRNAs) are a kind of endogenic, single-stranded non-coding RNA with about 19–23 nucleotides (8). This kind of RNA mainly exerts biofunction by binding to target genes, resulting in translational inhibition or degradation of target genes (9, 10). MiRNAs could regulate the initiation and progression of many kinds of tumors, including gastric cancer (11), colorectal cancer (12), and pancreatic cancer (13), etc. Emerging studies reported that miRNAs play a significant contribution to EC. For example, miR-31 is upregulated in esophageal squamous cell carcinoma (ESCC) tissues and serum samples, which is negatively associated with relapse-free survival of patients (14). The knockdown of miR-31 suppresses EC development by targeting Egln3 (14). Lower expression of miR-204-5p was observed in ESCC tissues and cell lines, its upregulation could inhibit proliferation, invasion, and promote apoptosis of ESCC cells (15). Meanwhile, miRNAs are also involved in the effect of chemotherapeutics. Upregulation of miR-338-5p reverses 5-Fluorouracil resistance in ESCC cells by targeting Id-1 (16). MiRNA-10b is upregulated in EC tissues and cells, which contributes to cisplatin resistance *via* targeting PPARγ (17).

MiR-135b-5p is an onco-miRNA. It is reported that miR-135b-5p is dysregulated, including gastric cancer cells and tissues, which promotes gastric cancer progression and metastasis through inhibiting CMTM3 expression (18). Similarly, miR-135b-5p was over-expressed in pancreatic cancer tissues, which represses lump growth by targeting phosphofructokinase-1 (19). In addition, miR-135b-5p expression is upregulated in ESCC patient samples (20). However, its exact mechanism and potential therapeutic potential in EC have not been fully clarified.

In order to investigate the role and mechanism for miR-135b-5p in EC. We confirmed it by experiments *in vivo* and *in vitro* and showed that miR-135b-5p is highly expressed in EC tissues and cells. Downregulation of miR-135b-5p suppresses EC progression by targeting TXNIP. Moreover, the miR-135b-5p/TXNIP axis also contributes to the anti-tumor effect of cisplatin in EC.

## Materials and Methods

### Bioinformatics Analysis

One hundred and sixty EC tissue samples and 11 normal esophageal tissue samples were collected from TCGA database (21). Kaplan-Meier Plotter (22) online tools were used to calculate the Kaplan-Meier survival analysis parameters.

### Cell Culture

In this study, the human EC cell lines (Eca109, KYSE150, EC9706) and normal esophageal epithelial cells (Het-1A) were acquired from the ATCC (Manassas, VA, USA). These cells were cultured in 1640 medium (RPMI; Gibco, CA, USA) and supplemented with 10% FBS and 1% penicillin-streptomycin (Gibco, CA, USA). HEK-293T cells were maintained in DMEM (Gibco, CA, USA) and added the same ingredients as above. These cells were cultured at 37°C under 5% CO2.

### Cell Transfection and Construction of Stable Cell Line

The synthesized miR-135b-5p mimics, anti-miR-135b-5p oligonucleotides (AMO-135b-5p), negative controls (miR-NC and AMO-NC) acquired from Ribo Life Science (GuangZhou, China). These syntheses and Lipofectamine 2000 reagent (Invitrogen, Carlsbad, CA, USA) were transfected into Eca109 and EC9706 cells. To explore the involvement of miR-135-5p in the effect of cisplatin (cis-diamminedichloroplatinum II, DDP), the Eca109 cells were treated with DDP (10 μg/ml) or phosphate buffer saline (PBS) respectively. To obtain stable cell lines, Eca109 was infected with the lentivirus particles contain miR-135b-5p (GenePhagma, Suzhou, China). Twenty-four hours after treatment, Eca109 cells were selected using 2 µg/ml puromycin. This procedure was repeated three times.

### Real-Time PCR

The RNA lysate (Invitrogen, Carlsbad, CA, USA) was used to obtain the total RNA from cells (Eca109, EC9706, KYSE150, Het-1A) and lump tissues (12). NanoDrop was used to measure RNA quality and quantity. RNA reversely transcribed into cDNA by using PrimeScript RT reagent kit (TOYOBO, Tokyo, Japan). A 7500 FAST real-time PCR System (Applied Biosystems, Carlsbad, CA, USA) was used to measure the amplified cDNA. The results were calculated using the 2^−ΔΔCT^ method. All primers were designed by Ribo Life Science (Guangzhou, China). All experiments were run in triplicate.

### Western Blot

Total protein from cells and tissues was extracted by RIPA (Beyotime, Shanghai, China). Protein suspension was quantified by BCA Protein Assay Kit (Beyotime, Shanghai, China). Ten percent SDS-PAGE and 12% SDS-PAGE (Beyotime, Shanghai, China) were used to separate protein lysate. And protein was then transferred to a PVDF membrane (Millipore, Boston, USA). The PVDF membrane was stained for Ponceau S and was closed with 5% skim milk powder for 2 h and incubated with antibodies against TXNIP, Bax, Bcl-2, and GAPDH (Proteintech, Wuhan, China) overnight. After washed by TBST, the PVDF membrane was incubated with the corresponding secondary antibody (Proteintech, Wuhan, China) for 2 h at room temperature and then washed again. GAPHD served as the internal control. ECL Plus by X-ray film (Millipore, Bedford, MA, USA) was analyzed the protein expression.

### Immunohistochemistry (IHC)

Four percent paraformaldehyde-fixed tissues were carried out with paraffin and sliced into 4 μm thick sections. Then 0.3% TritonX-100 penetrated tissues for 30 min and immunostained for Ki67, TXNIP (Proteintech, Wuhan, China) primary antibodies at 4°C overnight and universal secondary antibodies at 37°C for 60 min. Next, tissues were visualized by using the 3‐amino‐9‐ethylcarbazole was applied for 10 min. The tissues were washed with PBS for three times and tissues were counterstained with hematoxylin, then dehydrated and coverslipped according to the protocol.

### Cell Counting Kit-8 Assay (CCK-8)

The viability of Eca109 and EC9706 was assessed by the CCK8 assay (Meilun, Dalian, China). CCK- 8 assay 10 μl was joined in the 96-well plates at 0, 24, 48, 72, and 96 h after treatment, then cultured at 37°C for 1 h. The absorbance at 450 nm was measured by a microplate spectrophotometer (Bio-Rad, Hercules, CA, USA). Three independent experiments were performed.

### EdU Assay

The Proliferation ability of Eca109, EC9706 cell lines was also measured using an assay kit (Ribo Life Science, GuangZhou, China). Cells with transfected AMO-135b-5p or AMO-NC were seeded in 96-well plates (1 × 10^3^ cells/well) and incubated for 24 h. Cells were washed by PBS three times and cultured for 4 h in serum-free 1640 medium with 50 µM EdU. Then, the cells were fixed with 0.5% Triton-X-100 (Sigma-Aldrich, USA) for 30 min. After that, the cells were incubated with Apollo (Ribo life science, Guangzhou, China) staining reaction for 30 min. Finally, the Hochest was added to stain the nuclei of cells for 15 min. The percentage of Edu-positive cells was calculated by using Image-Pro-Plus software (Media Cybernetics, USA).

### Wound Healing Assay

Eca109 and EC9706 cells were transfected and cultured in a six-well culture plate to achieve 80% confluence. Subsequently, a straight line across the cell monolayer was drawn by a pipette tip (20 μl). After then, wash the cells use PBS to remove the debris. Twenty-four hours later, the images were captured by inverted microscopy. Relative cell migration distance was measured using Image-Pro software.

### Transwell Assay

Invasion capacity was measured with Matrigel matrix (Corning, NY, USA)–coated Transwell chambers (Corning, NY, USA). The matrigel matrix was dissolved at 4°C, then added to the upper chamber of the precooled transwell and incubated at 37°C for 2 h to solidify the matrigel matrix. Cells (1 × 10^5^) were cultured in serum-free medium and placed in the upper chamber of transwell, and cultural medium with 20% fetal bovine serum (FBS) was placed in the lower chamber. After cultured for 24 h, the transwell holes were penetrated by methanol solution and then staining by Crystal Violet. The images were taken at 200× magnification. The invaded cell number was counted in four random fields.

### Target Prediction and Dual-Luciferase Reporter Assay

The online tools miRTarBase (23), TargetScan (24), and DIANA-microT (25) predicted the complementary gene of miR-135b-5p. The wild-type (WT) and mutant-type (MUT) TXNIP 3’-UTR oligonucleotides miR-135b-5p were cloned into the psiCHECKTM-2 vector (Promega, Madison USA), which were transfected with miR-135b-5p mimic or AMO-135b-5p into HEK293T cells by Lipofectamine 2000 (Invitrogen, CA, USA) for 24 h.

### *In Vivo* Experiments

Six- to eight-week-old male nude BALB/c mice purchased from Animal Core Facility of Nanjing Medical University, Nanjing, China, and randomly divided into two groups (n = 5). Eca109 cells (5×106 per mice) with LV-has-miR-135b-5p-inhibitor vector (miR-135b-5p-inhibitor) or normal controls were injected into the subcutaneous back of mice. Mice body weight and tumor growth were recorded. Mice were sacrificed 4 weeks post-injection. The experiments were pre-approved by the ethics committee of the Second Affiliated Hospital of Harbin Medical University, NO. SYDW2020-067.

### Statistical Analysis

These statistical outcomes were provided as mean ± SD and calculated using GraphPad Prism 8.0. Student’s t-test was used to examine differences between two groups, while one‐way analysis of variance (ANOVA) was applied to compare the differences among multigroup. P<0.05 was set to have a statistical difference.

## Results

### The miR-135b-5p Highly Expressed in EC Tissues and Cells

**Figure 1:**
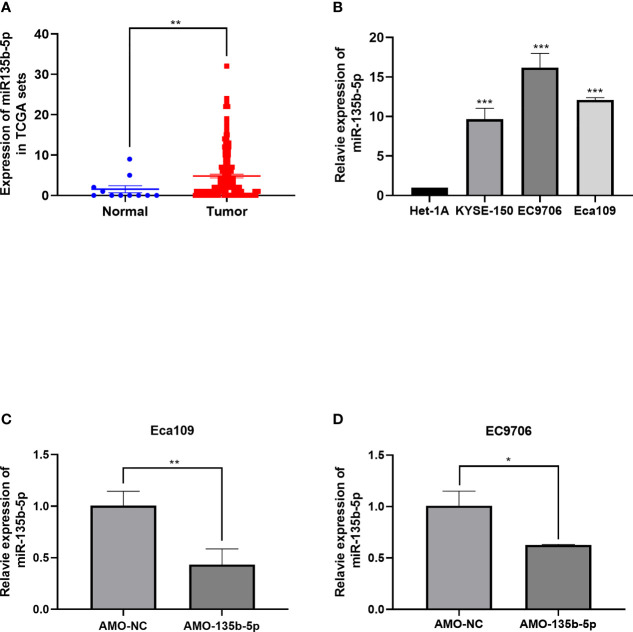
Expression of miR-135b-5p in EC tissues and EC cells. **(A)** MiR-135b-5p expression was higher in EC patient tissues than normal tissues. **(B)** MiR-135b-5p showed higher expression in Eca109, EC9706, KYSE150 cell lines than Het-1A cells. **(C, D)** Expression of miR-135b-5p with transfected AMO-135b-5p, AMO-NC are showed by real-time PCR. (*p < 0.05, **p < 0.01, ***p < 0.001).

MiR-135b-5p expression was increased in EC tissues (n = 160) compared with the normal samples (n = 11) (**Figure 1A**). Similarly, the miR-135b-5p expression was elevated in EC cells (Eca109, EC9706, KYSE150) compared with normal esophageal epithelium cell lines (Het-1A) (**Figure 1B**). We then explored the biological function for miR-135b-5p by transfecting AMO-135b-5p, AMO-NC, and control groups in EC9706 and Eca109 cells, respectively. MiR-135b-5p expression was reduced in Eca9706 and Eca109 cells after AMO-135b-5p transfection by using real-time PCR (**Figures 1C, D**). The comparison of the AMO-NC group and control group was added (**Supplementary Figure S1A**). These results observed miR-135b-5p expression was upregulated in EC tissues and cells. Eca109 and EC9706 cells showed a more significant increase of miR-135b-5p, and were used in the following experiments and showed the transfection efficiency successfully.

### Downregulated miR-135b-5p Effected EC Cell Proliferation and Apoptosis

**Figure 2:**
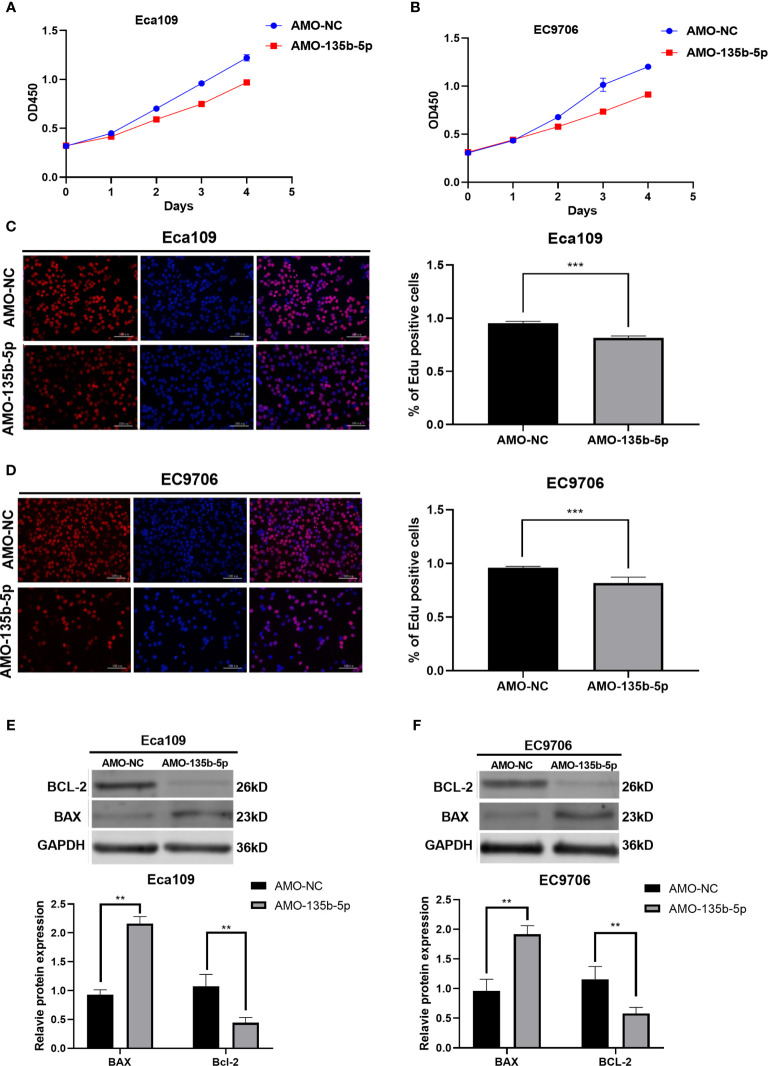
Downregulation of miR-135b-5p affected viability, proliferation, and apoptosis in Eca109 and EC9706 cells *in vitro*. **(A, B)** CCK8 assay showed the cell viability after with transfected AMO-135b-5p or AMO-NC in Eca109 and EC9706 cells. **(C, D)** Cell proliferation was measured by Edu analysis. Edu (red), nuclear (Hoechest, blue), magnification ×200. **(E, F)** The expression of Bax and Bcl-2 was showed by Western blot. Statistical analyses were showed accordingly. (**p < 0.01, ***p < 0.001).

CCK8 assay was used to evaluated EC cell viability. The administration of AMO-135b-5p suppressed cell viability in Eca109 and EC9706 cells compared to those transfected with AMO-NC (**Figures 2A, B**). In accordance with this result, the Edu assay showed that the number of proliferation cells in transfected AMO-135b-5p was less than the AMO-NC transfected groups in Eca109 and EC9706 cells (**Figures 2C, D**). Then, the expression of Bax and Bcl-2 antibodies reflected the apoptosis capacity. The results showed that the AMO-135b-5p group enhanced the expression of Bax, and decreased the expression of Bcl-2 than the AMO-NC group in Eca109 and EC9706 respectively (**Figures 2E, F**).

### Downregulated miR-135b-5p Suppressed EC Cell Migration and Invasion

**Figure 3:**
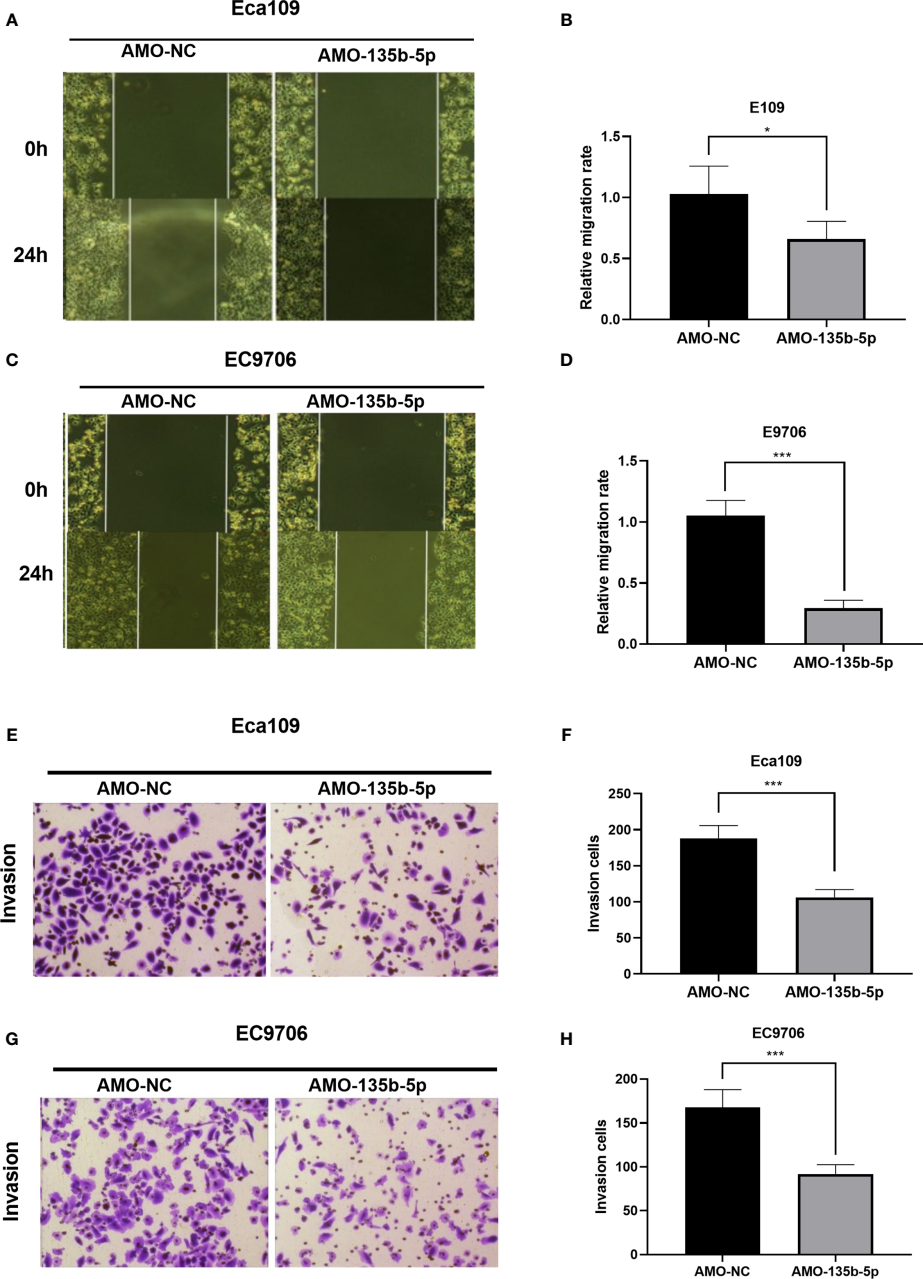
Downregulation of miR-135b-5p weakened migration and invasion ability in Eca109 and EC9706 cells. **(A–D)** Wound healing assay confirmed that miR-135b-5p inhibition restrained cell migration in Eca109 and EC9706 cells, statistical analyses were showed accordingly, magnification ×40. **(E–H)** Transwell assay evaluated that decreased miR-135b-5p expression abrogated invasion of Eca109 and EC9706 cells, magnification ×200. (*p < 0.05, ***p < 0.001).

The inhibition of miR-135b-5p weakens the migration ability of Eca109 and EC9706 cells compared to those in AMO-NC groups (**Figures 3A–D**). Then, the transwell assay analyzed the invasion ability in downregulated miR-135b-5p. Downregulated miR-135b-5p reduced the number of invasive cells relative to the negative control groups (**Figures 3E–H**).

### TXNIP Is the Complementary Gene of miR-135b-5p

**Figure 4:**
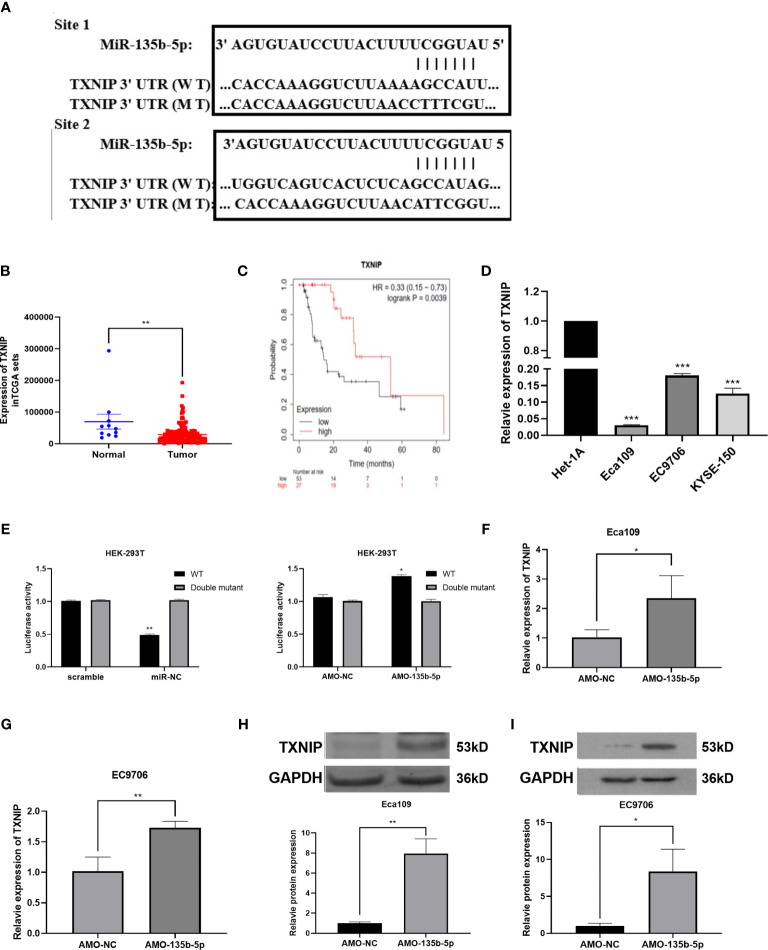
TXNIP as a complementary gene of miR-135b-5p. **(A)** TargetScan tool anticipated miR-135b-5p has two complementary positions with the 3’-UTR of TXNIP. **(B)** The expression of TXNIP in EC tissues. **(C)** KM plotter showed the relationship between TXNIP expression situation and the overall survival of EC patients. **(D)** TXNIP expression was significantly downregulated in Eca109, EC9706, KYSE150 cells than that in Het-1A cells. **(E)** Luciferase activities reporter assay analyzed the direct binding effect between miR-135b-5p and TXNIP. **(F–l)** Expression of TXNIP in with transfected AMO-135b-5p and AMO-NC, respectively (*p < 0.05, **p < 0.01, ***P < 0.001).

Bioinformatics prediction software [miRTarBase (23), TargetScan (24), DIANA-microT (25)] anticipated the complementary gene of miR-135b-5p. The result showed that the 3′-untranslated regions (3′-UTR) of TXNIP contain putative miR-135b-5p binding sequences (**Figure 4A**). TCGA data analyzed the TXNIP expression level was lower in EC tissues (n = 160) relative to normal samples (n = 11) (**Figure 4B**), and TXNIP expression was related to the survival rate of EC patients from the Kaplan-Meier Plotter (22). The results confirmed that compared with patients with lower TXNIP, patients with higher TXNIP had a better prognosis (**Figure 4C**). The real-time PCR assay demonstrated the TXNIP expression level was downregulated in the EC cells in comparison with that of Het-1A cells (**Figure 4D**). The binding site between miR-135b-5p and TXNIP was validated by a dual-luciferase reporter assay. Results showed that the miR-135b-5p mimics weakened the luciferase activity of TXNIP (**Figure 4E**). In accordance with this result, AMO-135b-5p transfection increased TXNIP expression in Eca109 and EC9706 cells (**Figures 4F, G**). Comparison of AMO-NC group and control group were showed (**Supplementary Figure S1B**). Western blot assay also demonstrated that AMO-135b-5p transfection elevated the expression of TXNIP (**Figures 4H, I**).

### The miR-135b-5p Inhibition Weakened Tumorigenicity *In Vivo*

**Figure 5:**
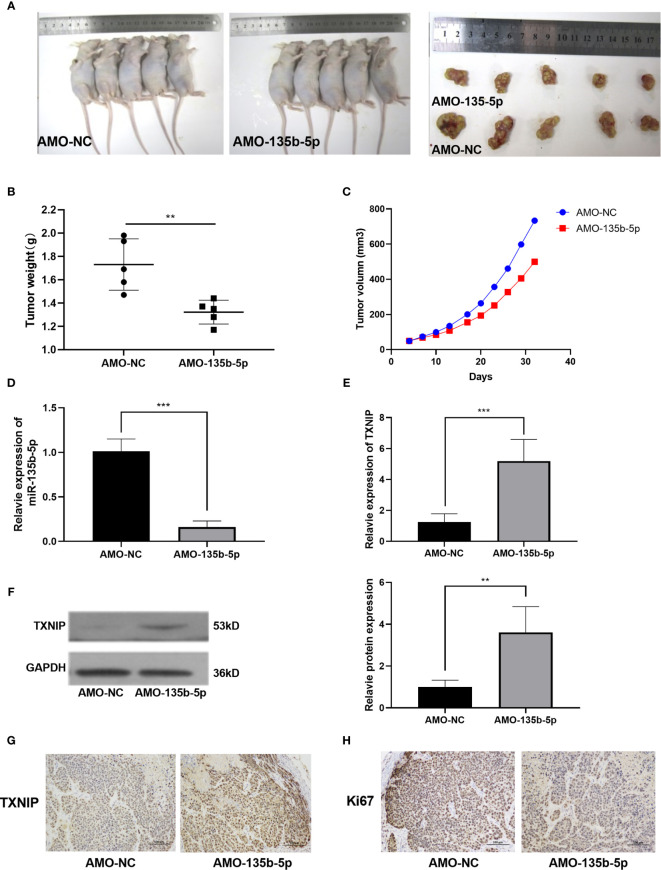
MiR-135b-5p inhibition suppressed the growth of EC *in vivo*. **(A)** Xenograft mice tumor model images on the 30th day of treatment. **(B)** The size of the tumors was smaller in the miR-135b-5p inhibition group compared with the negative control group. **(C)** The tumor growth curve was recorded. **(D, E)** The mRNA expression level of miR-135b-5p and TXNIP in miR-135b-5p inhibition group compared with the negative control group. **(F)** The protein expression level of TXNIP in miR-135b-5p inhibition group compared with the negative control group. **(G, H)** Immunohistochemistry of TXNIP and Ki67 expression in LV-miR-135b-5p and control, magnification ×200. **P < 0.01; ***P < 0.001.

Nude mice were administered with Eca109 cells (miR-135b-5p low expression or normal expression). The representative mice tumor images were shown in **Figure 5A**. The tumor growth curve showed that tumor growth was decreased by miR-135b-5p inhibition (**Figure 5B**). The tumor weight statistical analyses showed that tumor growth was slower in the miR-135b-5p inhibition group than the negative control (**Figure 5C**). MiR-135b-5p expression was downregulated and TXNIP was upregulated in the AMO-135b-5p group (**Figures 5D, E**). Western blot assay also demonstrated the TXNIP upregulated accordingly (**Figure 5F**). Similarly, the expression of Ki67 and TXNIP was analyzed by immunohistochemical assay. The results confirmed that the expression of Ki67 in the AMO-135b-5p group was increased, and the expression of TXNIP was decreased (**Figures 5G, H**).

### MiR-135b-5p/TXNIP Axis Participated in the Anti-tumor Effect of Cisplatin

**Figure 6:**
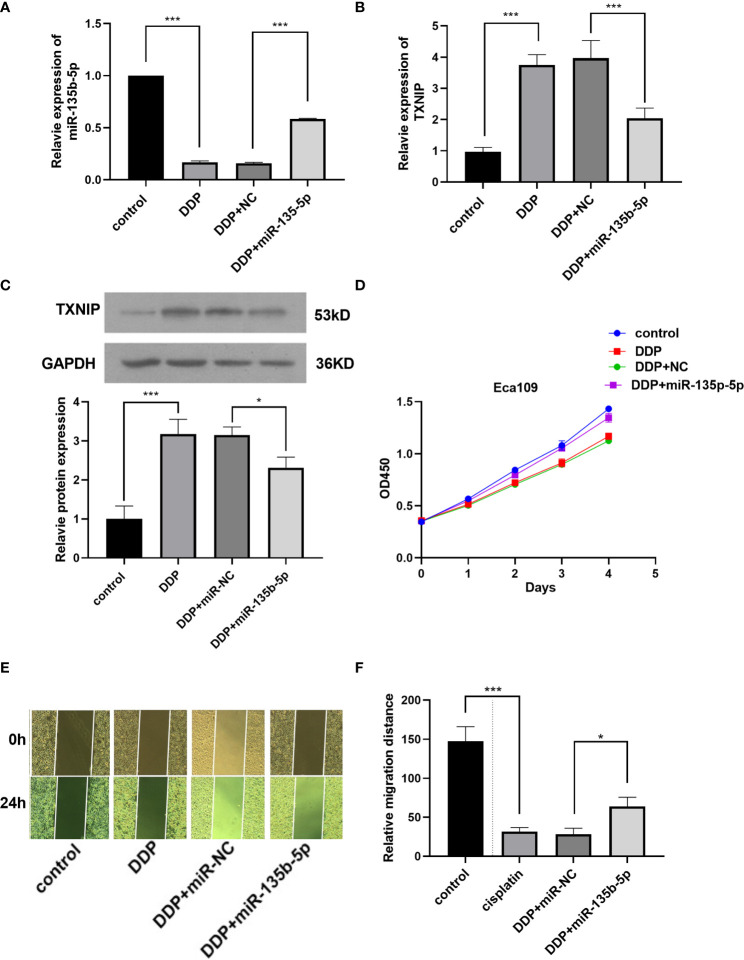
MiR-135b-5p/TXNIP axis contribute to the anti-tumor effect of cisplatin (DDP) for EC. **(A)** MiR-135b-5p expression level. **(B)** TXNIP mRNA expression level. **(C)** TXNIP protein expression level. **(D)** The miR-135b-5p mimics attenuated the inhibitory effect of DDP on the viability of Eca109 cells. **(E, F)** The miR-135b-5p mimics weakened the inhibitory effect of DDP on the migration ability of Eca109 cells. (*p < 0.05, ***P < 0.001).

**Figure 7:**
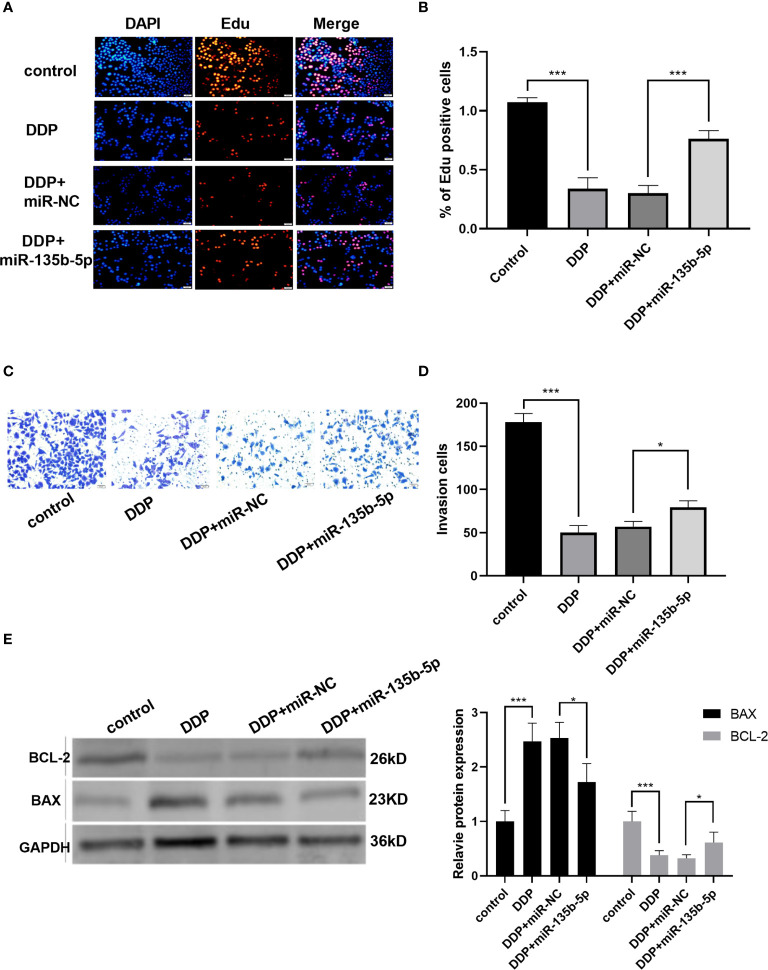
MiR-135b-5p/TXNIP axis contributes to the anti-tumor effect of cisplatin (DDP) for EC. **(A, B)** Cells proliferation was measured by Edu analysis. Edu (red), nuclear (Hoechest, blue), magnification ×200. **(C, D)** Transwell assay evaluated the invasion capacity after the intervention of DDP or DDP-mimics, DDP+miR-135b-5p magnification ×200. **(E)** Bax and Bcl-2 protein expression level and statistics were showed accordingly. (*p < 0.05, ***p < 0.001).

Finally, we investigated whether miR-135b-5p/TXNIP axis is engaged in the anti-tumor effect of DDP. DDP decreased miR-135b-5p expression and enhanced TXNIP expression in Eca109 and EC9706 cells. However, miR-135b-5p mimic transfection elevated the miR-135b-5p expression and weakened the TXNIP expression in two types of cell lines (**Figures 6A, B**). The protein expression level of TXNIP was changed accordingly (**Figure 6C**). The CCK8 assay evaluated the cell viability, DDP combined miR135b-5p mimics decrease the viability of Eca109 cells compared with that treated with DDP alone (**Figure 6D**). Besides, the upregulation of miR-135b-5p weakened the effect of DDP on the migration capacity of Eca109 and EC9706 cells (**Figures 6E, F**). Next, we also conducted the Edu assay to compare the proliferation capacity in Eca109 and EC9706 cells. The results showed that DDP significantly inhibited cell growth, and after mimics-135b-5p, the ability to suppress proliferation was limited (**Figures 7A, B**). Transwell assay showed the effect of DDP on tumor cells invasion inhibition after transfected mimics-135b-5p. It was showed that mimics-135b-5p decreased the effect of DDP (**Figures 7C, D**). Similarly, we carried out a western blot experiment to detect apoptotic proteins (Bax, Bcl-2). Lastly, we found that the Bax protein with treated DDP and DDP-NC group increased and Bcl-2 protein was decreased, while the mimic-135b-5p group attenuated the apoptosis effect of DDP (**Figure 7E**).

## Discussion

It is widely confirmed that miRNAs are involved in the progression of various cancers, which include bladder carcinomas, pancreatic cancer, and hepatocellular carcinomas, etc. (26, 27). Hammouz RY et al. reviewed the role of miRNAs in metastasis, angiogenesis phenotypes in bladder carcinomas (28). Recent research demonstrated that miR-1224-5p is a prognostic biomarker in colorectal cancer (12), which identified the important role of miRNA in different tumors. Some miRNA-based therapeutics have entered clinical trials (29). A miR-34a mimic (MRX34) has reached phase I clinical trials for cancers (30–32). The function and therapeutic potential of miRNAs have been identified. MiR-143 and miR-145 mimics have tumor-suppressive function in colon cancer and pancreatic cancer (31), which has entered the preclinical model. MiR-200 family has been reported with tumor-suppressive effect in solid tumors, involving breast, ovarian, and lung cancer (33–35). Several studies have reported that many kinds of miRNAs play a crucial role in the progression in EC, including miR-216a-5p and miR-488-3p, and miR-301b, etc. (36–38). These miRNAs may serve as novel therapeutic targets for EC. However, the role of aberrantly expressed miRNAs in EC has not been fully clarified.

Li W et al. found that miR-135b-5p is upregulated in ESCC tissues, which may be an indicator of shorter overall survival of patients (20). Our results evaluated miR-135b-5p is upregulated in Eca109 and EC9706 cells and its inhibition weakened proliferation, invasion, migration, and enhanced the apoptosis capacity. Our results are in accordance with previous researches. Wu Y et al. demonstrated that silencing miR-135b-5p attenuated the progression of gastric cancer (39). Zhou J et al. demonstrated miR-135b also had higher expression in pancreatic cancer stem cells and tissues. Silencing miR-135b-5p suppressed stemness of pancreatic cancer stem cells by targeting JADE-1 (40).

MiRTarBase (23), TargetScan (24), and DIANA-microT (25) predicted the complementary gene of miR-135b-5p. The prediction results showed that TXNIP could be a target of miR-135-5p. TXNIP is a kind of thioredoxin (TRX) binding protein, which mediates oxidative stress, inhibits cell proliferation, and induces apoptosis, and participated in a metabolic pathway, inflammatory pathway, and apoptotic pathway by inhibiting the function of the thioredoxin system in various diseases (41, 42). Increasing evidence demonstrated that TXNIP was a tumor suppressor and was shown a low expression in liver cancer, breast cancer, and lung cancer (41, 43). Morrison JA et al. validated that higher-expression of TXNIP significantly suppressed the growth of T238 cells and reduced metastasis of thyroid carcinoma in a mouse model (44). TXNIP overexpression weakened the progression of SMMC7221 cells by promoting ROS generation and activating MAPK pathway (45). It has been reported that TXNIP was significantly downregulated in EC cells (46). We identified that low expression of TXNIP was associated with a poor survival rate of EC patients. Besides, TXNIP expression level was lower in EC cells compared with that in normal esophageal epithelial cells. Subsequently, we explored the correlation with miR-135b-5p and TXNIP. Dual-luciferase reporter showed that miR-135b-5p binds to the 3′UTR of TXNIP directly. And, the inhibition of miR-135b-5p increased TXNIP expression.

Next, we established a subcutaneous tumor model by injection of Eca109 cells, with AMO-NC and AMO-135b-5p. The miR-135b-5p inhibition weakened tumor growth and increased the expression of TXNIP reversely. These results were in accordance with the *in vitro* experiments. MiR-135b-5p inhibition suppressed the progression of EC through targeting TXNIP.

Finally, we explored the therapeutic role of miR-135b-5p/TXNIP signaling in EC. Zhou J et al. observed that miR-135b-5p inhibition weakens the DDP resistance in gastric cancer cells (47). Ko M et al. discovered that a higher level of miR-135b-5p was associated with shorter median disease-free survival of patients than those with low (48). The present results implied that miR-135b-5p contributed to outcomes of DDP-treated patients. DDP inhibited miR-135b-5p expression and promoted TXNIP product in Eca109 and EC9706 cells. The enhanced expression of miR-135b-5p attenuated the inhibitory effect of DDP on the proliferation and migration of EC cells. These results proved that miR-135b-5p/TXNIP axis was engaged in the anti-tumor effect of DDP.

In conclusion, miR-135b-5p inhibition suppresses the progression of EC through targeting TXNIP. And the inhibition of miR-135b-5p/TXNIP axis might be a promising strategy to increase the anti-tumor effect of DDP. The above results could provide new insights into the investigation and treatment of EC.

## Data Availability Statement

The raw data supporting the conclusions of this article will be made available by the authors, without undue reservation.

## Ethics Statement

The animal study was reviewed and approved by the ethics committee of the Second Affiliated Hospital of Harbin Medical University.

## Author Contributions

YD and SJ conceived and designed this study and critically revised the article. YD and YJ performed the main experiments and wrote the manuscript. XYS, YG, and HC assisted with the xenograft model construction in mice. JL, XYS, DN, JJL, and BL assisted with the statistical analysis of data and drafted the literature review. SJ supervised the overall research, secured funding, and interpreted results. All authors contributed to the article and approved the submitted version.

## Funding

This project was supported by the National Natural Science Foundation of China (81803524 and 81803012), Harbin Medical University Fund for Distinguished Young Scholars.

## Conflict of Interest

The authors declare that the research was conducted in the absence of any commercial or financial relationships that could be construed as a potential conflict of interest.
