# Corrigendum: Downregulation of miR-135b-5p Suppresses Progression of Esophageal Cancer and Contributes to the Effect of Cisplatin

**DOI:** 10.3389/fonc.2021.785363

**Published:** 2021-11-15

**Authors:** Yuzhu Di, Yanan Jiang, Xiuyun Shen, Jing Liu, Yang Gao, Huimin Cai, Xiaoli Sun, Dandan Ning, Bing Liu, Jiaji Lei, Shizhu Jin

**Affiliations:** ^1^ Department of Gastroenterology and Hepatology, The Second Affiliated Hospital of Harbin Medical University, Harbin, China; ^2^ Department of Pharmacology (State-Province Key Laboratories of Biomedicine- Pharmaceutics of China, Key Laboratory of Cardiovascular Research, Ministry of Education), College of Pharmacy, Harbin Medical University, Harbin, China; ^3^ Translational Medicine Research and Cooperation Center of Northern China, Heilongjiang Academy of Medical Sciences, Harbin, China; ^4^ Department of Thoracic Surgery, The Second Affiliated Hospital of Harbin Medical University, Harbin, China

**Keywords:** esophageal cancer, miR-135b-5p, thioredoxin interacting protein, cisplatin, proliferation

In the original article, there was a mistake in ***Figure 1A*** and ***Figure 1B*** as published. The location of **Figure 1A** **and** **Figure 1B** **is incorrect**. The corrected ***Figure 1*** appears below.

**Figure 1:**
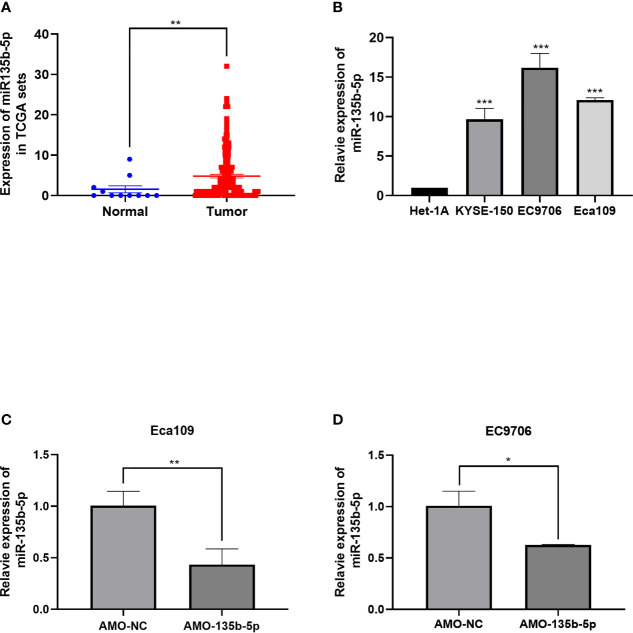
Expression of miR-135b-5p in EC tissues and EC cells. **(A)** MiR-135b-5p expression was higher in EC patient tissues than normal tissues. **(B)** MiR- 135b-5p showed higher expression in Eca109, EC9706, KYSE150 cell lines than Het-1A cells. **(C, D)** Expression of miR-135b-5p with transfected AMO-135b-5p, AMO-NC are showed by real-time PCR. (*p < 0.05, **p < 0.01, ***p < 0.001).

In the original article, there was an error. **Matrigel matrix was pre-coated to the upper chamber for 4°C overnight and then cells 1×10^5^ cultured in serum-free medium were placed in the upper chamber of transwell, and 20% serum-free medium were placed in the lower chamber.**

A correction has been made to ***MATERIALS AND METHODS Transwell AssayParagraph 1***

**“The matrigel matrix was dissolved at 4°C, then added to the upper chamber of the pre-cooled transwell and incubated at 37°C for 2 h to solidify the matrigel matrix. Cells (1 × 10^5^) were cultured in serum-free medium and placed in the upper chamber of transwell, and cultural medium with 20% fetal bovine serum (FBS) was placed in the lower chamber”**

The authors apologize for these errors and state that this does not change the scientific conclusions of the article in any way. The original article has been updated.

## Publisher’s Note

All claims expressed in this article are solely those of the authors and do not necessarily represent those of their affiliated organizations, or those of the publisher, the editors and the reviewers. Any product that may be evaluated in this article, or claim that may be made by its manufacturer, is not guaranteed or endorsed by the publisher.

