# Supplementary figures and images for: Downregulation of miR-135b-5p Suppresses Progression of Esophageal Cancer and Contributes to the Effect of Cisplatin
Source: Front Oncol. 2021 Jul 1;11:679348. doi: 10.3389/fonc.2021.679348 (PMC8281352; doi:10.3389/fonc.2021.679348)

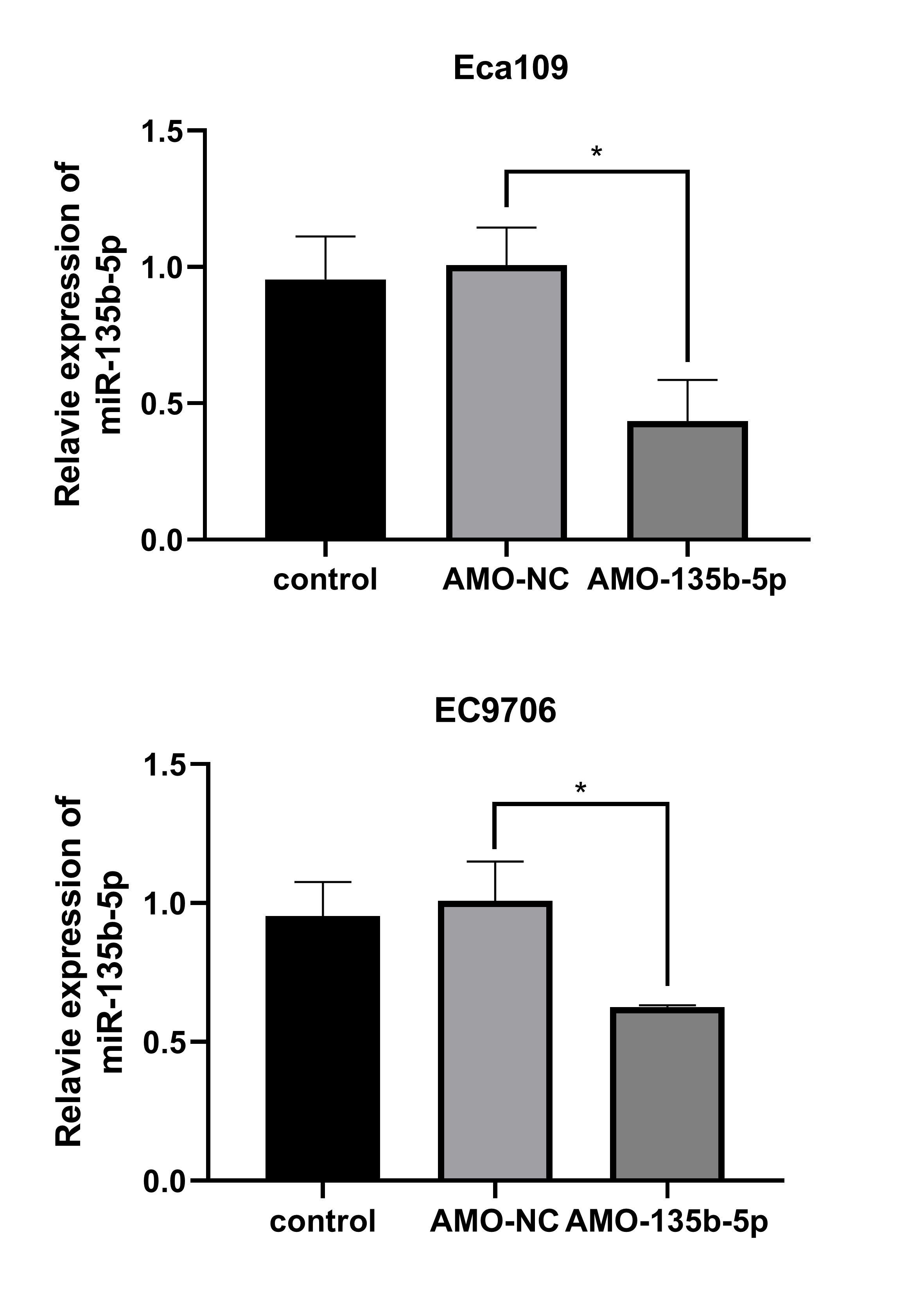

Supplement: Supplementary file 1 [file Image_1.tif]
